# An algorithm for Morphological Phylogenetic Analysis with Inapplicable Data

**DOI:** 10.1093/sysbio/syy083

**Published:** 2018-12-11

**Authors:** Martin D Brazeau, Thomas Guillerme, Martin R Smith

**Affiliations:** 1 Department of Life Sciences, Imperial College London, Silwood Park Campus, Buckhurst Road, Ascot SL5 7PY, UK; 2 Department of Earth Sciences, Natural History Museum, Cromwell Road, London SW7 5BD, UK; 3 School of Biological Sciences, The University of Queensland, St. Lucia 4067, Queensland, Australia; 4 Department of Earth Sciences, University of Cambridge, Downing Street, Cambridge CB2 3EQ, UK; 5 Department of Earth Sciences, Mountjoy Site, Durham University, South Road, Durham DH1 3LE, UK

**Keywords:** Character independence, character optimization, cladistic analysis, inapplicable data, phylogenetic tree search

## Abstract

Morphological data play a key role in the inference of biological relationships and evolutionary history and are essential for the interpretation of the fossil record. The hierarchical interdependence of many morphological characters, however, complicates phylogenetic analysis. In particular, many characters only apply to a subset of terminal taxa. The widely used “reductive coding” approach treats taxa in which a character is inapplicable as though the character’s state is simply missing (unknown). This approach has long been known to create spurious tree length estimates on certain topologies, potentially leading to erroneous results in phylogenetic searches—but pratical solutions have yet to be proposed and implemented. Here, we present a single-character algorithm for reconstructing ancestral states in reductively coded data sets, following the theoretical guideline of minimizing homoplasy over all characters. Our algorithm uses up to three traversals to score a tree, and a fourth to fully resolve final states at each node within the tree. We use explicit criteria to resolve ambiguity in applicable/inapplicable dichotomies, and to optimize missing data. So that it can be applied to single characters, the algorithm employs local optimization; as such, the method provides a fast but approximate inference of ancestral states and tree score. The application of our method to published morphological data sets indicates that, compared to traditional methods, it identifies different trees as “optimal.” As such, the use of our algorithm to handle inapplicable data may significantly alter the outcome of tree searches, modifying the inferred placement of living and fossil taxa and potentially leading to major differences in reconstructions of evolutionary history.

Morphological characters are an essential source of data in phylogenetic studies. Even in the age of molecular sequence data, they underpin a range of research programs that depend on knowledge of extinct or ancestral phenotypic conditions (e.g., palaeontology, molecular clock calibrations, and comparative developmental biology). Despite advances in the use of probabilistic models for analyzing morphological data (Lewis 2001; Wright et al. 2016), all transformation-based methods (e.g. parsimony and likelihood) are subject to a common and persistent problem: not all characters in a data set logically apply to all taxa under consideration. This problem arises due to hierarchical relationships between characters.

Maddison (1993) famously showed that treating character state inapplicability as missing data—still one of the most popular approaches to handling inapplicable data—is prone to artifactual tree length calculations that could misdirect phylogenetic searches. The essence of the problem is that existing parsimony methods measure the amount of homoplasy—the true target metric of parsimony (De Laet 2005)—by calculating the total number of character transformations. However, this only works if character states refer exclusively to properties of homologous structures (Platnick 1979). To date, popular software for phylogenetic analysis has failed to account for this problem, which leaves open the question of whether existing computational methods are even appropriate for morphological data sets that incorporate character hierarchies.

Numerical phylogenetic methods require a 2D matrix of character state scores for a set of terminal taxa under investigation. This requires the construction of either a molecular sequence alignment, or a character list and table of morphological trait values. “Transformation-based” phylogenetic methods (e.g., parsimony, maximum likelihood, or Bayesian inference) treat each individual column (character or transformation series) in the matrix as independent, and use the states in each column to calculate the score of the tree.

However, it is easy to show that characters in both morphological and molecular data sets can exhibit nonindependence. Logical character dependence (Wilkinson 1995a) manifests as hierarchical relationships between morphological characters, where a character that scores an attribute of a feature presupposes the presence of that feature. In the case of molecular sequence data, this is most commonly seen in the case of gaps, which presumably arise from insertion or deletion events (indels; for example, the character “nucleotide at position X” is not applicable if position X does not exist in one taxon due to deletion). In either case, some characters in the data set can only have a meaningful value for a subset of the species under investigation.

The process of encoding characters in a 2D matrix and summing their implied transformations under the assumption of independence introduces a problem. Hierarchical character relationships themselves contain information, which might be ignored: indels, for instance, represent evolutionary events and therefore provide phylogenetic information. This has opened up research into techniques for dynamic (or direct) alignment of sequence data (Sankoff 1975; Wheeler 1995, 1996, 1999; De Laet 2005; Liu et al. 2009; Varón et al. 2010; Liu et al. 2012; De Laet 2015) in which alignment is co-optimized with tree reconstruction, either simultaneously or in phases. The case of morphology can be considered a special case of the more generalized problem (De Laet 2005, 2015), but has nevertheless seen fewer attempts to address it. In this article, we focus on the special case of morphological character hierarchies.

## The Computational Problem of Morphological Character Hierarchies

In current programs for phylogenetic analysis, an investigator has the choice to treat inapplicability as either a state of its own or as missing data. This allows for numerous ways to “atomize” character variables in a matrix, each with different mathematical (and theoretical) implications which have been extensively explored and reviewed (Farris 1988; Platnick et al. 1991; Maddison 1993; Pleijel 1995; Wilkinson 1995a; Strong and Lipscomb 1999; Hawkins 2000; Fitzhugh 2006; Brazeau 2011). Arguably the most popular method of dealing with character hierarchies is to use a reductively coded neomorphic transformation series (*sensu*Wilkinson 1995a; Sereno 2007; Brazeau 2011) to denote the presence or absence of a principal character, and one or more ontologically dependent transformation series (which may employ a coding approach somewhere on the spectrum from reductive to composite coding) to denote attributes of the principal character:

1. Tail: absent (0); present (1)
2. Tail color: blue (0); red (1)

In the event that a taxon is scored 0 for character 1, then it will be treated as having missing data in character 2. This approach is hoped not to lead to implicit (and unintended) character weighting, but does entail spurious calculations. Because the subsidiary character (here, “tail color”) is assigned a state at every node, situations exist in which a logically impossible transformation may be reconstructed (e.g., a change in tail color in an ancestor with no tail; see Vignette Section 2, Maddison 1993). These logically impossible state reconstructions and their concomitant transformations have been informally referred to as “pseudo-parsimony,” but could be generalized to “pseudo-optimality,” since they would occur in probabilistic calculations as well. Maddison (1993) showed that this can distort the scores of individual trees and consequently misdirect phylogenetic searches.

In spite of the problem of logically impossible state reconstructions, this coding strategy is still widely used, and is generally viewed as the most appropriate approach (Strong and Lipscomb 1999; Brazeau 2011; but see also arguments from Fitzhugh 2006; Vogt 2018). This is because, unlike other methods, it is seen as least likely to discard useful phylogenetic information or to accumulate redundant changes (see above references for a discussion of these problems). The challenge, therefore, is to create algorithms that “understand” the difference between inapplicable and missing data. In this article, we review some of the practical and theoretical questions of morphological character hierarchies and propose a single-character parsimony algorithm. We also present a $\texttt{C}$ library and an $\texttt{R}$ (R Core Team 2017) package that can be used to conduct phylogenetic tree searches with this new algorithm.

## Limitations of Sankoff Matrices

Forey and Kitching (2000) showed that it is possible to express character hierarchies in terms of Sankoff matrices. They advocated this as a tenable solution to the problem of inapplicable data. If this were the case, then there would be no real need to solve the problem of morphological character hierarchies at an algorithmic level. However, mathematical and practical limitations of the Sankoff approach render it undesirable for phylogenetic analysis.

The primary mathematical problem is that the Sankoff method may result in severe overestimation of the number of losses, in proportion to the number of substates in the character (see Vignette Section 2.5 for an illustration). This is because the matrix creates an imbalance in favor of losses, regardless of how much (or how little) additional pairwise homology is implied between any two branches.

From a practical perspective, each new character combination requires the calculation and storage of an individual cost for each possible combination of the state of that character and the states of other characters encoded in the same Sankoff matrix. The addition of a single character can greatly increase the computational time required to optimize the tree. Desirable practical properties of new algorithms and programs would—where possible—avoid this level of complexity and be readily applied to existing data matrices without the need for substantial recoding.

## Theoretical Background for Parsimony with Character Hierarchies

It has recently been shown that the “inapplicable data” problem cannot be simply reduced to the calculation of the number of evolutionary transformations in single-column characters (De Laet 2005, 2015). In a parsimony framework, the quantity being minimized is the amount of homoplasy, in reference to Hennig’s auxiliary principle that assumptions of non-homology are to be minimized. De Laet (2005, 2015) argues that, more precisely, parsimony is based on a preference for maximized pairwise homology, the justification being that this maximizes the amount of pairwise similarity that is explained by the tree. This is consistent with the general justification of parsimony, which seeks to minimize the amount of homoplasy.

**Figure 1.**
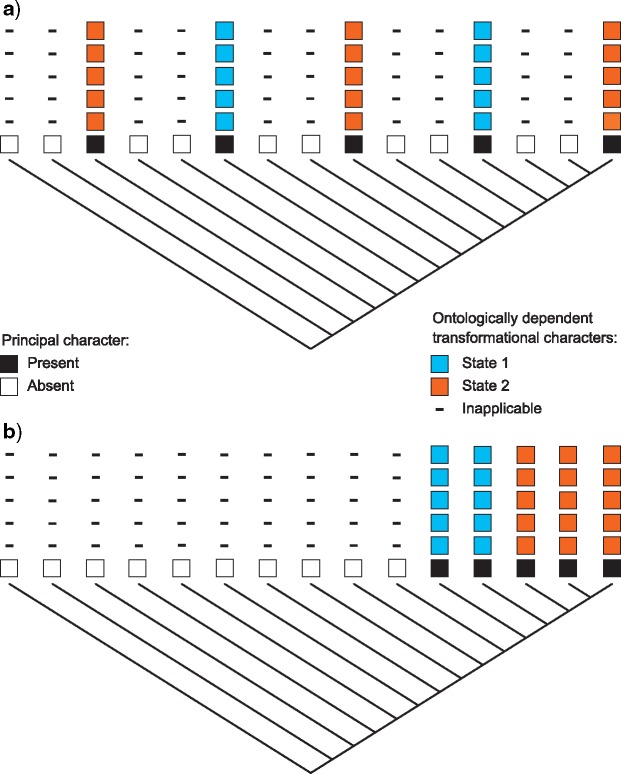
Effect of counting method on tree preference. If no cost is given for transitions between the inapplicable symbol and any applicable states, transformations between losses and gains of the principal character will be inadequately penalized, then trees with multiple gains of the principal character (a) will be favored; if transformations between applicable and inapplicable states are penalized, then trees in which the principal character evolves exactly once (b) will be favored.

In reductively coded data sets, there is no clear way to count “steps” on a tree when inapplicable data are involved. Although it would be tempting to simply assign no cost to transformations involving the inapplicable symbol, this will not work. This is most clearly illustrated in the context of a principal character with a number of hierarchically dependent transformational characters (Fig. 1; see Vignette Section 2.6). If transformations between applicable and inapplicable states contribute nothing to tree length, then independent appearances (e.g. gains) of a character have no added cost from subordinate characters, even when they are identical (i.e., share putative homology). This can, in some cases, result in a penalty for character congruence (Fig. 1), and thus a penalty for homology: a situation we consider inconsistent with the theory of phylogenetic parsimony. To again borrow Maddison’s (1993) example, a single transformation from “tail absent” to “tail present, red” does not represent an instance of homoplasy for the ontologically dependent character “tail color.” However, if this same transformation happens twice, homoplasy in tail color has occurred. Thus, the tree should be penalized once for the independent origin of the second tail, and once more because the second tail, when it appeared, happened to exhibit the same state (red) as the first (see Vignette Section 3.1). In contrast, the loss of a tail implies the simultaneous loss of color and other similar attributes, which cannot similarly be explained as transformations.

*Scoring trees*.— In presenting a solution to the problem of scoring trees, De Laet (2005, 2015) introduces the concept of a subcharacter or, equivalently, regions of character applicability. Homology can be maximized by co-minimizing the number of transformations and the number of regions (subcharacters). The logic behind this is not obvious, but is fairly simple. When inapplicables are present, a variable character can either transform at least once (e.g., a change from red to blue) or be split into two or more subcharacters (i.e., two separate appearances of tails). If tails can be either present or absent in an analysis, and have two possible colors (red and blue), then splitting tails into three clades involves at least five homoplasious observations: three independent tail origins, plus at least two clades that have independent appearances of exactly the same tail color.

Throughout this manuscript we therefore make a clear distinction between tree *length* and tree *score*. Tree length designates the number of transformational events (steps) implied by a topology, whereas tree score designates an optimization value that can combine some function of the tree length with other nontransformational events, such as the sum of the number of applicable regions.

## Single-Character Parsimony with Inapplicable Data

*Preliminary assumptions*.—Here we describe our algorithm procedure using informal terms. A formal description of the algorithm using set logic is included in the Appendix. The following assumptions are made, consistent with the theory outlined above:

- The data set contains separate transformation series for the principal character and each contingent character.
- A separate signifier (usually “-” also called the “gap” symbol) has been used to denote that a character is inapplicable for a particular taxon.
- Missing data are equivalent to a polymorphism consisting of all possible states (applicable and inapplicable). It is possible, but rarely desirable (Vignette Section 5), to define missing data as “unknown, but must be from the set of applicable states.”

The state symbols used for logically applicable values are called “applicable states.” The state symbol used to denote inapplicability will be called the “inapplicable state.” We further assume that the distribution of applicable and inapplicable states in the dependent character will match the distribution of the principal character to which they are related.

*General principle*.— Given these latter assumptions and an accurately coded data set, our algorithm can be applied without any specification of which character in the data set is the principal character. This differs from De Laet’s (2005; 2015) method, which uses a prior specification of hierarchy.

Our algorithm attempts to reconstruct ancestral states where inapplicable values are present by first resolving the implicit distribution of “applicable” and “inapplicable” states. Then, it resolves any character state transitions between applicable tokens. Steps are counted for normal transitions. On the way down the tree, a tracker variable detects and records whether the character has been “split” into multiple regions of applicable tokens.

**Figure 2.**
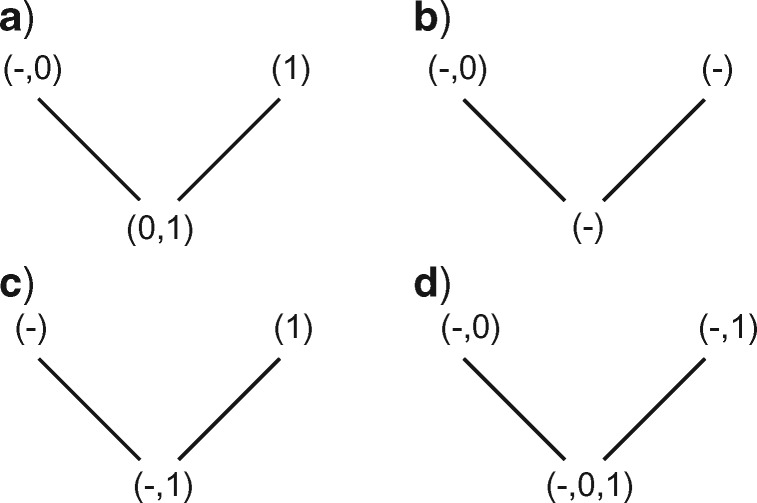
First downpass procedures. Where both descendants have an applicable state, but only one has the inapplicable state, reconstruct the node as having all of the applicable states (a); where both descendants have only the inapplicable state in common, the node is reconstructed with the inapplicable state only (b); where one descendant has only the inapplicable state, and the other has any applicable state(s), the node is reconstructed as all the applicable and inapplicable states (c); where both descendants have the inapplicable state, as well as applicable states, reconstruct the node as having all descendant states (d).

**Figure 3.**
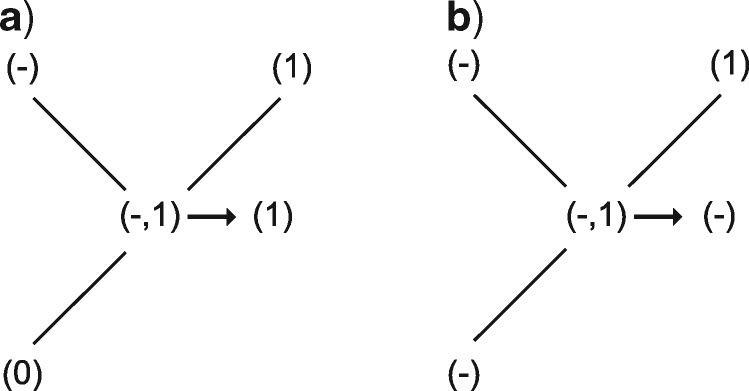
First uppass procedures. When two or more adjacent nodes are applicable, the node is applicable (a); otherwise, the node is inapplicable (b).

**Figure 4.**
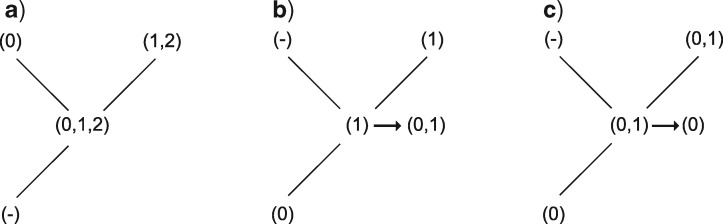
Second uppass procedures. If a node’s ancestor is inapplicable, its state requires no further modification (a). If a node contains applicable tokens and its ancestor has no states in common with its applicable descendant(s), the ancestral states are added to to the node’s (b); but if its ancestor and descendants do have states in common, these common states become the node’s states (c).

**Figure 5.**
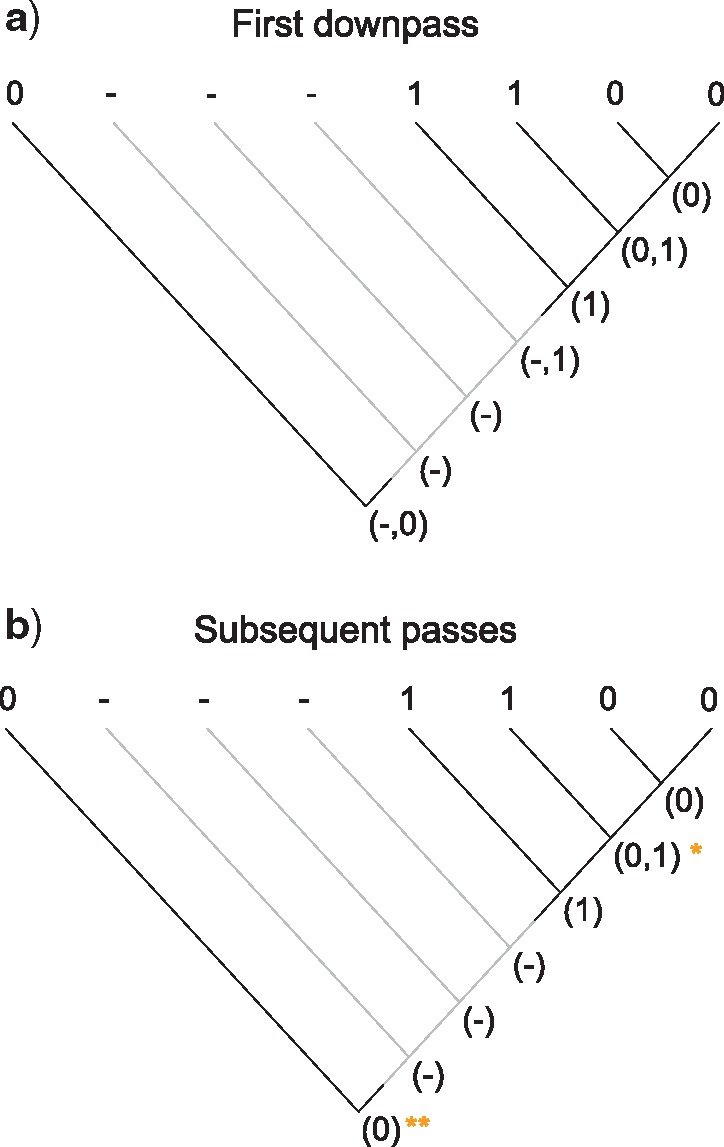
Scoring of a simple tree with inapplicable data. A principal character is present in two regions of the tree (black lines). A transformation from state 1 to state 0 adds one step to tree length. A second occurrence of state 0 represents a case of homoplasy and should also contribute to tree score. The first downpass of our algorithm (a) generates possible reconstructions of each node; the state reconstructions generated in the first uppass happen not to be modified by further passes. The second downpass calculates this character’s contribution (+2) to the tree’s score, reflecting one transformation (at *) and one additional region (at **).

**Figure 6.**
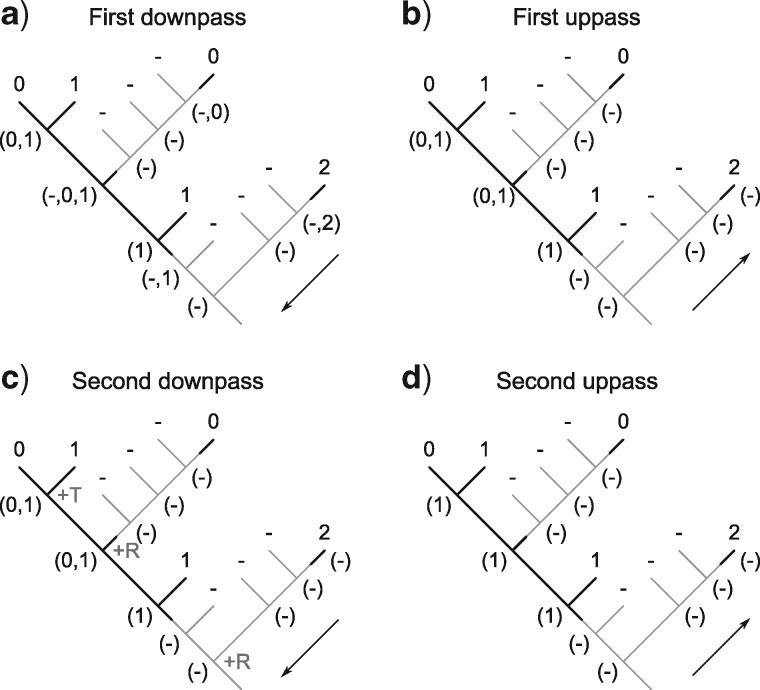
Our algorithm performs an initial downpass (a) and uppass (b) to assign each node to an applicable or inapplicable region. A second downpass (c) counts standard Fitch transformations (+T) and additional applicable regions (+R), the latter counted at nodes whose left descendant leads to a different applicable region than their right descendant.

To accomplish this, the algorithm proceeds in two sets of down- and up-passes on the tree (Figs 2–6): the first resolves regions of applicability and inapplicability, while the second resolves character state transformations within the regions of applicability. The following instructions apply to a single character at a single node, which is assumed to be binary and have a single ancestor (the root has no ancestor). An interactive visualization of the four passes is available via the $\texttt{Inapp}$$\texttt{R}$ package (Guillerme et al. 2018; https://github.com/TGuillerme/Inapp).

### First Procedure: Resolve Presence-or-Absence Status of Each Node

#### First downpass

1. If any descendant has any applicable states (regardless of whether they are the same state), but at most one descendant has the inapplicable state, the nodal set is constructed by combining all applicable states (i.e., without the inapplicable state, Fig. 2a).
2. Otherwise, if both descendants have the inapplicable state, but at most one descendant has an applicable state, reconstruct the nodal set as inapplicable (Fig. 2b).
3. Otherwise, combine all applicable and inapplicable states into the nodal set (Fig. 2c,d).

#### First uppass

Consider the three branches incident to the node being evaluated: two descendants and one ancestor. (In the case of the root node, the ancestor should be considered to have an applicable state.)

1. If two or more adjacent nodes have applicable states, reconstruct the nodal set as having only the applicable states (Fig. 3a).
2. Otherwise, the node is reconstructed as inapplicable (Fig. 3b).

We base this on a theoretically justifiable assumption: choose the reconstruction that assumes parallel losses over loss and regain—that is, choosing the presence over the absence of a character at any ambiguous node. Minimizing the number of independent origins in the neomorphic character is generally considered preferable, as it is more likely to preserve homology between similar complex characters (Agnarsson and Miller 2008, see Vignette Section 1.3.1). This has the benefit of speeding up execution of the algorithm. However, this differs from De Laets method; it is locally optimal, but there are cases where it may not be globally optimal.

### Second Procedure: Conditionally Resolve Applicable States and Score the Tree

In order to score the tree by counting the number of extra character regions, a second variable is introduced here: a ”tracker” that records whether or not any applicable regions exist on the subtree upwards of the given node. For computational simplicity, we use two logical states for the tracker: “true” when the region contains applicable states, and “false” when it contains the inapplicable state. All tips with applicable states have their tracker set to “true”; inapplicable tips are set to “false.”

After this stage of the algorithm is applied, each vertex must be unambiguously applicable or inapplicable. Ambiguous tips are resolved thus: if the ancestor is applicable, remove the inapplicable state from the terminal state set; if the ancestor is inapplicable, remove the applicable state(s) from the terminal state set.

#### Second downpass

The second downpass constructs the applicable state sets at nodes that have been resolved as applicable. It applies Fitch rules to descendant nodes if the current node is applicable. It is during this pass that the tracker values are updated, and the score of the tree is calculated.

1. If the node is applicable:
  (a) Check for states in common between descendant nodal sets. If there are states in common and they are applicable values, the preliminary nodal set is formed by the applicable states in common.
  (b) Otherwise, if there are no descendent states in common, but both descendants have applicable states, construct a set consisting of all descendant applicable states. *Add one step to the tree score*.
  (c) Otherwise, if at most one descendant has applicable states, add those states to the nodal set of the current node. Check the trackers of both descendants: if both indicate descendants with applicable regions, *add one region count to the tree score*.
2. Otherwise, if the node is in the inapplicable state, check the trackers of both descendants: if both indicate descendants with applicable regions, *add one region count to the tree score*.
3. Update the tracker: if any descendant tracker is “true,” set the current nodal tracker to “true.” Otherwise, it is set to “false.”

#### Second uppass

The second uppass finalizes the ancestral state estimations for the applicable states. It operates on the normal Fitch rules conditional to the prior resolution of only applicable values at this node.

If the current nodal set has only applicable states, follow normal Fitch rules, with the following exceptions:

1. If the immediate ancestor has only the inapplicable state, then the current nodal set is final (it is equivalent to the root of the tree for this character, and thus no further changes are required, Fig. 4a).
2. If at most one of the descendants is in the inapplicable state, add to the final nodal set any states in the ancestor not found in the descendant with applicable states (Fig. 4b).
3. If any applicable states are shared in common between ancestor and descendant, remove from the set any applicable states not found in both the descendant and the ancestor (Fig. 4c).

The complete optimization of characters proceeds in four passes: two sets of downpass–uppass traversals on the tree to calculate final ancestral state sets (Fig. 5). Three passes are therefore sufficient to calculate the score of any tree; four are required to reconstruct the character states at every node (Fig. 6).

We have been unable to identify a process that accomplishes this in two passes (as would be sufficient for a single, independent character under the Fitch algorithm). We believe that a two-pass method is impossible for realistic data sets (i.e., with ambiguity and missing data), though we have not proven this formally. Nevertheless, it stems from intuition that the number of passes required to simultaneously resolve *n* characters with a hierarchic relationship will likely require 2*n* passes on the tree, as each principal character needs to be fully resolved before satisfactory resolution of the next dependent character.

The formal description of this algorithm is available in the Appendix. The algorithm has been implemented in Shiny ($\texttt{R}$) (Guillerme et al. 2018) and $\texttt{C}$ (Brazeau et al. 2017; http://www.morphyproject.org/). Phylogenetic search using the $\texttt{C}$ implementation is implemented in the $\texttt{TreeSearch}$$\texttt{R}$ package (Smith 2018), available from the CRAN repository. An informal, step by step illustration and explanation of the algorithm is provided in Vignette Section 3.2.

## Properties of the Algorithm and Implications for Character Coding

### Effect of Minimizing Regions on Character Distributions in the Tree

The method of minimizing the number of independent character regions has important mathematical properties that distinguish it from the standard Fitch procedure. However, as with the Fitch algorithm, this algorithm is symmetrical, and thus gives the same final result and tree score regardless of the rooting of the tree. However, the following discussion assumes the tree is rooted in order to explore the evolutionary implications of the algorithm.

Under our algorithm, a tree incurs costs for transformations and costs for additional regions. Therefore, even an invariant subordinate character with inapplicability (i.e., consisting of only one applicable state, with some taxa inapplicable) will inform a phylogenetic search. An example would be a data set where some taxa do not have tails, and other taxa do have tails, all of which happen to be blue. Here, a cost is incurred for a gain or a loss of a tail, and an additional cost will be incurred every time an independent instance of blueness appears.

This has an advantage and a drawback. The advantage is that complex similarity between characters can be objectively weighted without increasing the penalty for losing that character. Unlike the case where “inapplicable” is treated as a character state, the cost of a loss is not compounded by summing nonindependent losses over all subordinate characters. The main drawback is that apparently uninformative characters that previously had no impact on the results might disproportionately penalize certain topologies. Care must be taken, therefore, that each hierarchically dependent character truly reflects a biologically significant similarity, for a principal character might be misleadingly upweighted if trivial subordinate properties (e.g., “number of distinct bases in tail DNA”) are included in a matrix.

### Hierarchies of Neomorphic Characters

It is important to distinguish between ontogenetically dependent characters (Vogt 2018) that are transformational (as in the case of tail color) and neomorphic (Sereno 2007). Up to this point, we have only dealt with an instance of a transformational character which happens to take one of two states, red and blue, whenever a tail is present. A neomorphic subcharacter, in contrast, refers to a presence-or-absence character that is subordinate to the principal character. In this case, it might be a structure such as an eyespot or spine that is situated on the tail itself. Our method opens up choices for how an investigator may wish to encode this information at the level of character definition and matrix construction. Each option has different mathematical consequences.

Table 1 shows two different ways of coding tail eyespots contingent on the presence of a tail. The first method is the reductive coding strategy. The second is equivalent to additive binary coding of Kluge and Farris (1969). The possible advantage of additive binary coding is that it will not lend support to clades that are united by the absence of eyespots (see Vignette Section 4.4.1). It can be argued that the absence of eyespots is a condition that conveys less information than presence of eyespots. On this basis, an investigator may wish to give increased importance to the presence of the eyespot, but no particular importance to its absence (unless there is a loss). In this case, additive binary coding will be preferable. The consequence of this approach, however, is that the loss of a tail requires two steps (one for the loss of the tail itself and a second for the loss of the eyespot). If this is considered unrealistic, then the investigator might prefer reductive coding.

**Table 1.** Coding inapplicable data in ontologically dependent characters. “Tail” is a principal character with two ontologically dependent characters:, “Tail color,” a transformational character that should be coded as “-” when a tail is absent; and “Tail eyespot,” a neomorphic character that should be coded as “0” when a tail is absent. “?” is used to denote ambiguity in cases where the presence of a tail is known, but its characteristics uncertain.

Tail	0	0	0	1	1	1	1	1	(0, absent; 1, present)
Tail color	-	-	-	0	0	1	2	?	(0, red; 1, blue; 2, green; -, inapplicable)
Tail eyespot (reductive coding)	-	-	-	1	0	1	0	?	(0, absent; 1, present)
Tail eyespot	0	0	0	1	0	1	0	?	(0, absent; 1, present)
(additive binary coding)									

## Comparing Approaches to Phylogenetic Reconstruction

In order to evaluate whether the treatment of inapplicable data meaningfully impacts phylogenetic results, we analyzed 30 discrete morphological matrices (Smith et al. 2018) under three approaches: (i) treating inapplicable tokens as missing data (the “missing” approach); (ii) the “extra state” approach, treating inapplicability as a separate character state; and (iii) the “inapplicable” approach, which applies our new algorithm.

Before beginning our analysis, matrices were inspected to confirm that their coding followed the assumptions made by our algorithm (by checking whether gaps and missing data symbols were defined separately and whether both symbols occurred in the matrices), and every inapplicable token in each neomorphic character was replaced with the token corresponding to the presumed nonderived condition (typically “absent”), following the additive binary coding approach advocated above. Each matrix was then subjected to phylogenetic tree search: the “missing” and “extra state” approaches used TNT, employing the parsimony ratchet, sectorial search, and tree drifting algorithms (Goloboff 1999; Goloboff and Catalano 2016); the “inapplicable” approach used the parsimony ratchet, implemented in $\texttt{TreeSearch}$ 0.0.8 (Smith 2018). Because it is difficult to guarantee that every optimal tree will be identified, we ensured a wide sampling of tree space in TNT by conducting 100 independent tree searches, and in $\texttt{R}$ by sampling shortest trees until the shortest length had been found by 250 ratchet iterations.

In order to establish whether the three methods recovered different sets of optimal trees, we tallied the number of distinct bifurcating trees that occurred in the optimal sets of one, two, or all three approaches. In addition, we calculated a strict consensus tree for all bifurcating trees in each optimal set, the number of bipartitions present in each set serving as a proxy for the disparity of trees that are optimal under each approach. Finally, each set of optimal trees was plotted in a 2D space (Hillis et al. 2005) by decomposing a matrix of pairwise quartet distances (Estabrook et al. 1985), calculated using the $\texttt{tqDist}$$\texttt{R}$ library (Sand et al. 2014), into two dimensions by minimizing the Kruskal-1 stress function (Borg and Groenen 2005), following Hillis et al. (2005).

## Results

**Figure 7.**
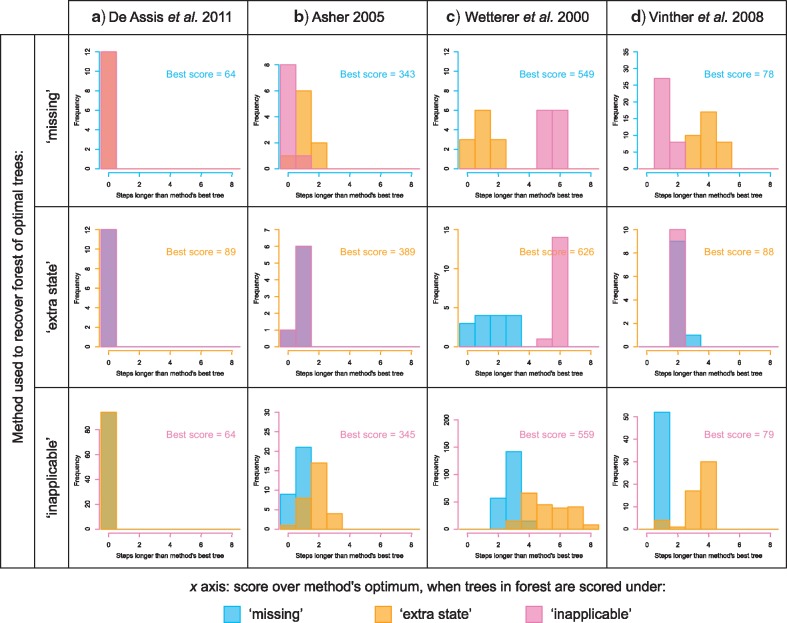
Different methods recover different optimal tree sets. Each histogram details the distribution of tree scores when each of the optimal trees recovered under method P is scored using method Q. Scores are presented relative to the lowest score recovered by method Q for each data set. Histograms for all examined data sets are presented in the Supplementary material.

**Figure 8.**
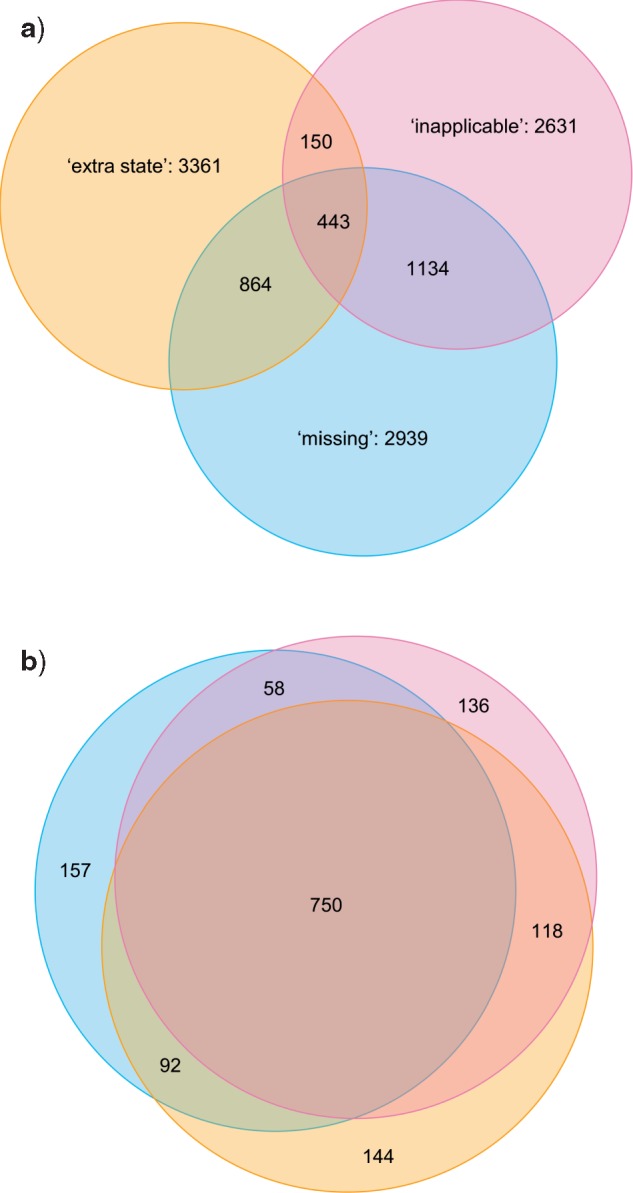
Venn diagrams depicting (a) proportions of optimal trees that are optimal under one, two, or three methods; b) proportion of nodes present in every optimal tree recovered under one, two, or three methods. Results are summed across all data sets; figures for individual data sets are available in the Supplementary material.

In most cases, the three different methods identified different sets of optimal trees. Indeed, only in one of the 30 examined data sets were the optimal trees recovered by each method also optimal under the other two (Fig. 7a). In 10 data sets (Fig. 7b), a subset of trees are optimal under all methods, but other trees are optimal under one method and a few steps longer under another. In nine data sets (Fig. 7c), the forests of trees that are optimal under two methods (here, “missing” and “extra state”) partially overlap, but in one method (here, “inapplicable”), no optimal trees were found that are also optimal under either other method. In the final 10 data sets (Fig. 7d), each method generates a distinct set of optimal trees. Summing across all data sets, only 4% of trees that were optimal under one method were also optimal under the other two (Fig. 8a).

**Figure 9.**
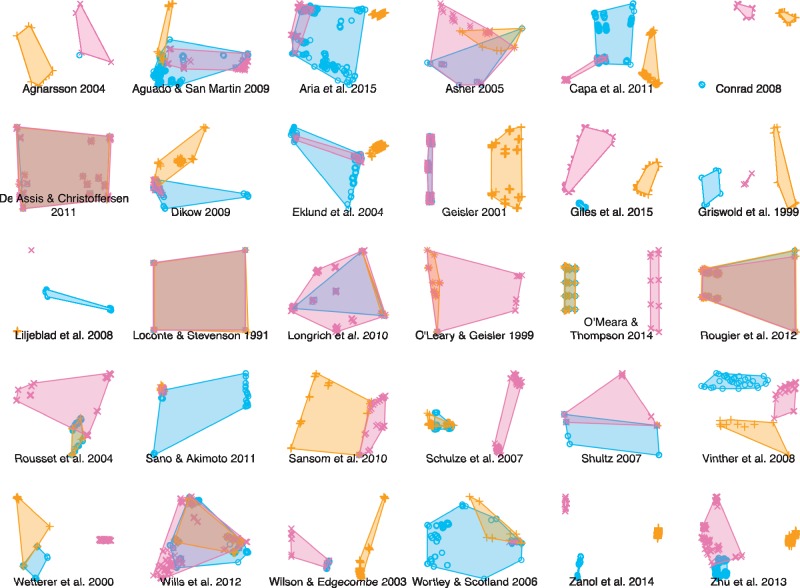
Distribution of optimal trees in MDS treespace for each data set. Shaded regions correspond to convex hulls surrounding all optimal trees recovered using a given approach. No method is consistently more precise or more similar to any other method.

How topologically different were the trees that each method described as optimal? One qualitative way to explore the difference between multiple forests of trees is to generate a 2D treespace from the distances between pairs of trees. This approach demonstrates that it is difficult to predict which methods will identify the most similar sets of optimal trees, and that the regions of treespace identified as optimal by the different methods may be very different or very similar (Fig. 9).

An alternative way to explore how much trees in the three optimal sets have in common is to count the number of bipartitions held in common between trees within a set—or, in other words, the number of bipartitions present on the strict consensus of all trees in that set. On this approach, averaged across all data sets, 76% of the bipartitions that are present in every tree that is optimal under the “inapplicable” approach are also present in every tree that is optimal under the “missing” approach, and 82% are present in every tree that is optimal under the “extra state” approach; only 70% are present in all trees recovered by all methods (Fig. 8b).

Even though, in any one data set, the number of trees identified as optimal can vary considerably between the three methods, we were unable to identify any systematic trend in the disparity of optimal trees. Neither the number of distinct trees in the optimal tree set, nor the resolution of the strict consensus tree, nor the area of treespace occupied by the trees showed any systematic variation with respect to properties of the underlying data sets.

## Conclusion

We have presented a single-character modified Fitch algorithm for ancestral state reconstructions that is aware of a special “inapplicable” token. This algorithm avoids logically impossible reconstructions of ancestral states by acknowledging that applicable state distributions rely on the prior resolution of dichotomies between applicable and inapplicable characters.

Because applicable state assignments depend on the resolution of the outcome of this dichotomy, up to four passes may be required to correctly calculate tree length. Furthermore, missing data need to be updated at the tips—initially as either applicable or inapplicable—in order to complete ancestral state sequences.

Our tree scoring procedure follows De Laet (2005) in penalizing increasing amounts of homoplasy without redundant penalties, but differs in that each character reconstruction must be locally parsimonious. Up to three traversals are necessary in order to score a tree, whereas a final reconstruction of character states requires a fourth traversal (a second uppass). The method, unsurprisingly, takes additional time, though this is expected to be mostly in proportion to the number of characters having inapplicable tokens. Nevertheless, some economies are possible, because only characters with three or more inapplicable tokens need to be treated with this algorithm. The method provides a means of evaluating existing data sets with minimal modification, and without a need to specify explicit relationships between characters (because the presence or absence of a parent character is already implicit in the distinction between applicable and inapplicable states). Preliminary results show that analyses with nontrivial amounts of inapplicable data are likely to be considerably influenced by mishandling of inapplicable data. In some cases, the set of trees that are optimal under our new algorithm does not overlap with the optimal sets obtained by standard Fitch parsimony, indicating that the effects on inapplicable data on morphological data sets have been substantial. Further work will be necessary in order to compare the results of these analyses with those of exact methods (such as De Laet’s forthcoming anagallis program), assess accuracy using meaningful simulations, and extend these approaches for use with explicitly probabilistic methods.

## Implementations

The algorithm described throughout this paper is implemented at different levels in different projects. The main $\texttt{C}$ implementation of the algorithm and associated tools is available at http://www.morphyproject.org/ (Brazeau et al. 2017). Phylogenetic search using the $\texttt{C}$ implementation available in the $\texttt{TreeSearch}$$\texttt{R}$ package (Smith 2018), available from the CRAN repository or https://github.com/ms609/TreeSearch. Finally, a shiny ($\texttt{R}$) visualization of the algorithm is available via the $\texttt{Inapp}$ package at https://github.com/TGuillerme/Inapp (Guillerme et al. 2018).

## Appendix

### Description of Algorithm

#### Definitions

Recognizing that this algorithm uses terminology from disparate fields, we define key terms as follows:

Our algorithm is applied to a bifurcating tree: a connected graph in which all vertices are either order 1 (referred to as tips), order 3 (nodes), or, in the single case of the root node, order 2. The tree is directed: it is a representation of evolutionary history, in which the basal (root) node is taken to represent the branching point that corresponds to the earliest branching point in the lineages’ shared evolutionary history. Each node except the root thus has one ancestral node—its immediate neighbour in the direction of the root—and two descendant nodes.

A token represents the possible presence of a particular character’s state at a particular point on a tree. The user specifies which tokens may occur at each tip; the algorithm identifies all tokens that may be present at each tip and each node, subject to the constraint that the token distribution maximizes homology. Each applicable token corresponds to a user-specified state of the coded character; the inapplicable token denotes that the coded character does not apply. If the applicability of the character is ambiguous at a vertex, then it will bear the inapplicable token in addition to one or more applicable tokens.

The tokens present at a particular vertex are considered to represent a set. The union of two sets of tokens contains all tokens that are present in either set: for example, the union of $\{\hbox{-}0\}$ and $\{01\}$ is $\{\hbox{-}01\}$.

A traversal of the tree involves visiting each vertex in a specified order. A postorder traversal starts at the tips, and only visits a node once all its descendants have been visited; a preorder traversal works from the root, and only visits vertices once their ancestor has been visited.

### First Postorder Traversal (Downpass)

Traverse the internal nodes of the tree in postorder (Figs. 2, 5a; Vignette Section 3.2.1). At each node:

1. **If** there is any token in common between both descendants, *go* to 2; **else***go* to 3.
2. **If** the token in common is only the inapplicable token, and both descendants have an applicable token, *set* the node’s state to be the union of the descendants’ states; **else***set* the node’s state to be the token in common between both descendants. **Then***go* to 4.
3. **If** both descendants have an applicable token, *set* the node’s state to be the union of both descendants’ states without the inapplicable token; **else***set* the node’s state to be the union of its descendants’ states. **Then***go* to 4.
4. Visit the next node in postorder. Once all nodes have been visited, *conduct the first uppass*.

### First Preorder Traversal (Uppass)

Traverse the tree in preorder (Figs. 3, 5b; Vignette Section 3.2.1). At each node:

1. **If** the node has the inapplicable token, *go* to 2; **else** leave the node’s state unchanged and *go* to 8.
2. **If** the node also has an applicable token, *go* to 3; **else***go* to 4.
3. **If** the node’s ancestor has the inapplicable token, *set* the node’s state to be the inapplicable token only and *go* to 8; **else***remove* the inapplicable token from the current node’s state. **Then***go* to 8.
4. **If** the node’s ancestor has the inapplicable token, *set* the node’s state to be the inapplicable token only and *go* to 8; **else***go* to 5.
5. **If** any of the descendants have an applicable token, *set* the node’s state to be the union of the applicable states of its descendants; **else***set* the node’s state to be the inapplicable token only. **Then***go* to 8.
6. **If** the unvisited tip includes both inapplicable and applicable tokens, *go* to 7; **else***go* to 8
7. **If** the current node has only the inapplicable token, *set* the tip’s state to the inapplicable token only; **else***remove* the inapplicable token from the tip’s state. **Then***go* to 8.
8. **If** one of the node’s descendants is an unvisited tip, *go* to 6; **else** visit the next node in preorder. Once all nodes and tips have been visited, *initialise the tracker*.

### Initialise Tracker

Visit each tip in turn (Fig. 5b, Vignette Section 3.2.2.1). At each tip:

1. **If** the tip’s state contains the inapplicable token, *set* its tracker to “false” and *go* to 4; **else***go* to 2.
2. **If** the tip’s state does not contain the inapplicable token, *set* its tracker to “true” and *go* to 4; **else***go* to 3.
3. **If** the ancestor’s state contains an inapplicable token, *set* the tip’s tracker to “false”; **else***set* the tip’s tracker to “true.” **Then***go* to 4.
4. Visit the next tip. Once all tips have been visited, *conduct the second downpass*.

### Second Postorder Traversal (Downpass)

Traverse the tree in postorder (Fig. 5b; Vignette Section 3.2.2). At each node:

1. **If** the tracker of either descendant is “true,” *set* this node’s tracker to “true”; **else***set* it to “false.” **Then**, *go* to 2.
2. **If** the node had an applicable token in the first uppass, *go* to 3; **else***go *to 7.
3. **If** there is any token in common between both descendants, *go* to 4; **else***go* to 5.
4. **If** the tokens in common are applicable, *set* the node’s state to be the tokens held in common, without the inapplicable token; **else***set* the node’s state to be the inapplicable token. **Then***go* to 9.
5. *Set* the node’s state to be the union of the states of both descendants (if present) without the inapplicable token, and *go* to 6.
6. **If** both descendants have an applicable token, ***add one to*** the tree score (denoting a transformation from one applicable state to another) and *go* to 9; **else***go* to 7.
7. **If** both of the node’s descendants’ trackers are “true,” ***add one to*** the tree score (denoting a previously uncounted region) and *go* to 9; **else***go* to 8.
8. **If** the tracker of both descendants is “true” **and **the node has only the inapplicable token, ***add one to*** the tree score (denoting a previously uncounted region) and *go* to 9; **else** just *go* to 9.
9. Visit the next node in postorder. Once all nodes have been visited, *report the tree score*. If character state reconstructions are required at all nodes, *conduct the second uppass*.

### Second Preorder Traversal (Uppass)

Traverse the tree in preorder (Figs. 4 and 5b; Vignette Section 3.2.3). At each node:

1. **If** the node has any applicable token, *go* to 2; **else***go* to 9.
2. **If** the node’s ancestor has any applicable token, *go* to 3; **else***go* to 9.
3. **If** the node’s state is the same as its ancestor’s, *go* to 9; **else***go* to 4.
4. **If** there is any token in common between the node’s descendants, *go* to 5; **else***go* to 6.
5. *Add* to the current node’s state any token in common between its ancestor and its descendants and *go* to 9.
6. **If** the states of the node’s descendants both contain the inapplicable token, *go* to 7; **else***go* to 8.
7. **If** there is any token in common between either of the node’s descendants and its ancestor, *set* the node’s state to be its ancestor’s state; **else***set* the current node’s state to be all applicable tokens that are common to both its descendants and ancestor. **Then***go* to 9.
8. *Add* to the node’s state the tokens of its ancestor. **Then***go* to 9.
9. Visit the next node in preorder.

## Vignettes

This paper is accompanied by a series of vignettes that aim to expound relevant principles in a clear and extended fashion. These supplementary materials are archived on GitHub (https://github.com/TGuillerme/Inapp/releases/tag/v0.4.1), (permanent archive) and are best viewed in HTML format (short URL: https://goo.gl/WFsX9d).
